# Association between Phosphatase Related Gene Variants and Coronary Artery Disease: Case-Control Study and Meta-Analysis

**DOI:** 10.3390/ijms150814058

**Published:** 2014-08-13

**Authors:** Xia Han, Lijun Zhang, Zhiqiang Zhang, Zengtang Zhang, Jianchun Wang, Jun Yang, Jiamin Niu

**Affiliations:** 1 Department of Cardiology, Laiwu People’s Hospital, Laiwu 271100, China; E-Mails: lwhx197984@126.com (X.H.); lijunzhan99@126.com (L.Z.); zhangzhiqian19@126.com (Z.Z.); ZhangZengtang@126.com (Z.Z.); 2 Department of Cardiology, Shandong Provincial Hospital, Jinan 250000, China; E-Mail: jianchunwan345@163.com; 3 Department of Cardiology, Yantai Yuhuangding Hospital, Yantai 264000, China; E-Mail: yangju20099@yeah.net

**Keywords:** coronary artery disease, SNP, *PTPN11*, *PHACTR11*, *ACP1*, meta-analysis

## Abstract

Recent studies showed that the serum alkaline phosphatase is an independent predictor of the coronary artery disease (CAD). In this work, we aimed to summarize the association between three phosphatase related single nucleotide polymorphisms (rs12526453, rs11066301 and rs3828329) and the risk of CAD in Han Chinese. Our results showed that the rs3828329 of the *ACP1* gene was closely related to the risk of CAD in Han Chinese (OR = 1.45, *p* = 0.0006). This significant association of rs3828329 with CAD was only found in the females (Additive model: OR = 1.80, *p* = 0.001; dominant model: OR = 1.69, *p* = 0.03; recessive model: OR = 1.96, *p* = 0.0008). Moreover, rs3828329 was likely to exert its effect in females aged 65 years and older (OR = 2.27, *p* = 0.001). Further meta-analyses showed that the rs12526453 of *PHACTR11* gene (OR = 1.14, *p* < 0.0001, random-effect method) and the rs11066301 of *PTPN11* gene (OR = 1.15, *p* < 0.0001, fixed-effects method) were associated with CAD risk in multiple populations. Our results showed that the polymorphisms rs12526453 and rs11066301 are significantly associated with the CAD risk in multiple populations. The rs3828329 of *ACP1* gene is also a risk factor of CAD in Han Chinese females aged 65 years and older.

## 1. Introduction

Coronary artery disease (CAD) is one of the leading causes of death in the developing and developed countries [1]. CAD is a complex disease which involves a variety of genetic and environmental factors [2]. Many CAD susceptibility loci have been identified [3], though it is believed that perhaps 95% or more of genes involved in the pathogenesis of CAD are yet to be clarified [4]. Among the proposed mechanisms, phosphatase regulation is likely to play a specific role in vascular development in CAD progression [5].

Three genetic variants connected with phosphatase are shown to be associated with the risk of CAD [6,7], including rs12526453 of *PHACTR1*, rs3828329 of *ACP1* and rs11066301 of *PTPN11*. The *PHACTR1* gene encodes the protein phosphatase 1 and actin regulator 1 (PHACTR1), an enzyme regulating endothelial nitricoxide in humans [8]. PHACTR1 has been demonstrated to be an important modulator in the pathophysiology of cardiovascular disease [9], and it may be involved in the formation of stenosis in cardiac vessels of CAD [10]. Acid phosphatase locus 1 (ACP1) is a member of the phosphotyrosine protein phosphatase family of proteins and is involved in metabolic signaling [11], growth signaling [12], immunological diseases [13] and cancer development [14]. ACP1 controls the synthesis of an enzyme involved in important metabolic functions [15]. ACP1 may participate in immune responses involved in the pathogenesis of atherosclerosis [16]. Previous study has found that high ACP1 activity could enhance the signaling from T cell antigen receptors and aggravate local coronary inflammatory lesions [15]. As a member of the protein tyrosine phosphatase (PTP) family, protein tyrosine phosphatase non-receptor type 11 (PTPN11) is encoded by the *PTPN11* gene and is known to modulate multiple signaling involved in inflammatory responses [17,18]. PTPN11 can positively regulate endothelial cell motility and angiogenesis [19], and increased PTPN11 expression may possibly accelerate aortic atherosclerosis [20]. PTPN11 plays an important role in a variety of diseases, such as atherosclerosis [21], glioma [22], myeloproliferative neoplasms [23] and gastric cancer [24,25]. Activating *PTPN11* mutations have also been detected in acute myeloid leukemia [26], breast cancer [27], colorectal cancer [28], and CAD [29]. However, there is no published study focused on the association between the three SNPs and CAD risk in Han Chinese.

This study aimed to summarize the contribution of the polymorphisms of three genes (*PHACTR1*, *ACP1* and *PTPN11*) to the CAD risk in Eastern China by meta-analyses and case-control study. 

## 2. Results

### 2.1. Literature Analysis

**Figure 1:**
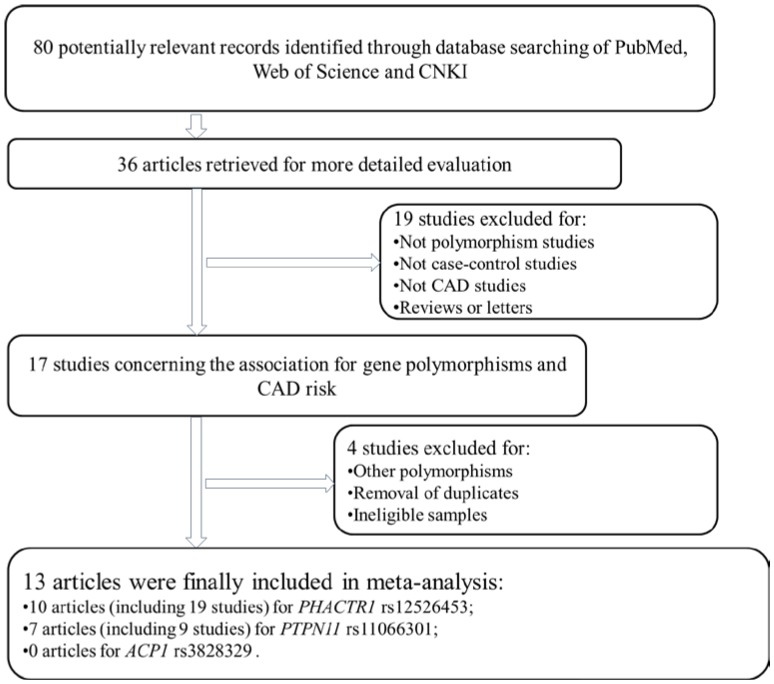
Flow diagram depicts literature search and study selection.

As shown in Figure 1, 36 studies were considered potentially eligible after a detailed screening of 80 potentially relevant studies from PubMed, Wed of Science and CNKI. After reading the title or abstract, 36 studies were considered for the following screen. Then 19 studies were excluded since they were not polymorphism or case-control studies, or were other cardiovascular studies, reviews or letters. Among the 17 studies remaining, 4 studies with nonrelevant SNPs or duplicates were removed. Finally, 13 studies that focused on the relationship between rs12526453 or rs11066301 polymorphisms and CAD risk were collected. However, no articles were focused on rs3828329 of the *ACP1* gene.

### 2.2. Meta-Analysis Results

**Figure 2:**
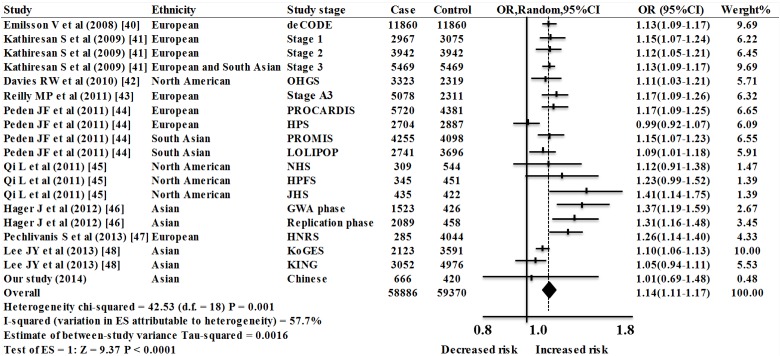
Meta-analysis of 19 association studies between rs12526453 and risk of CAD. Stage 1: MIGen (Myocardial Infarction Genetics Consortium); Stage 2: WTCCC (Wellcome Trust Case Control Consortium), GerMIFSI (German MI Family Study I), PennCATH (PennCATH), Medstar (MedSTAR); Stage 3: AMI Gene (Acute Myocardial Infarction Gene Study/Dortmund Health Study), Verona (Verona Heart Study), MAHI (Mid-America Heart Institute), IFS (Irish Family Study), GerMIFSII (German MI Family Study II), INTERHEART; OHGS (Ottawa Heart Genomics Study); Stage A3: Emory GeneBank, Utah Intermountain Heart Collaborative Study, Verona Heart Study; PROCARDIS (Precocious Coronary Artery Disease), HPS (Heart Protection Study); PROMIS (Pakistan Risk of Myocardial Infarction Study); LOLIPOP (London Life Sciences Prospective Population); NHS (Nurses’ Health Study), HPFS (Health Professional Follow-Up Study), JHS (Joslin Heart Study); HNRS: Heinz Nixdorf Recall Study; KoGES: Korea Genome Epidemiology Study; KING: Kita Nagoya Genome.

**Figure 3:**
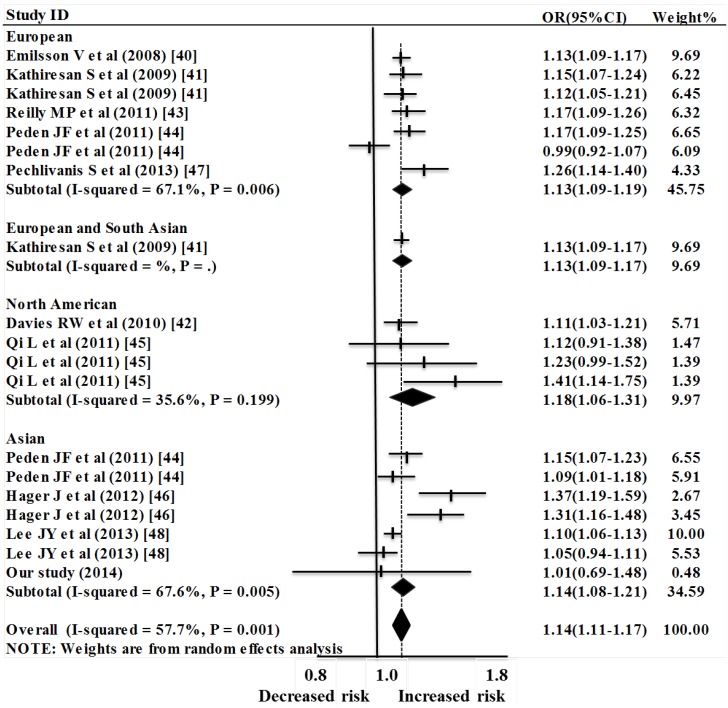
Meta-analysis of the association between rs12526453 and CAD risk stratified by population.

A total of 10 reports including 19 studies (including 58,886 CAD cases and 59,370 controls) were selected in the meta-analysis for the association of rs12526453 (*PHACTR11*) with CAD. Since substantial heterogeneity was observed among the overall studies (*p* = 0.001, I^2^ = 57.7%), a random-effect model was applied for the meta-analysis. As shown in Figure 2, the data showed that rs12526453 of the *PHACTR11* gene was a risk factor for CAD (overall OR = 1.14, 95% CI = 1.11–1.17, *p* < 0.0001, random-effect method). The analysis was carried out in multiple populations including European, North American, and Asian. Strong association of rs12526453 with CAD was observed in the European studies (OR = 1.14, 95% CI = 1.09–1.19, P(z) < 0.0001), the North American studies (OR = 1.18, 95% CI = 1.06–1.31, P(z) = 0.002) and the Asian studies (OR = 1.14, 95% CI = 1.08–1.21, P(z) < 0.0001). Subgroup analysis indicated that the population was likely to be the main source of heterogeneity (Figure 3). Specifically, large heterogeneities were found in the European studies (I^2^ = 67.1%, *p* = 0.006) and the Asian studies (I^2^ = 67.6%, *p* = 0.005). In contrast, no heterogeneity was observed in the North American studies (I^2^ = 35.6%, *p* = 0.199).

**Figure 4:**
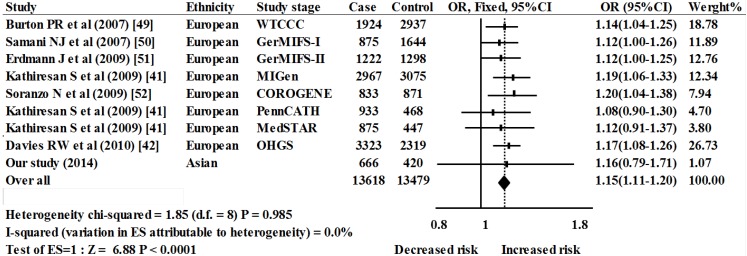
Meta-analysis of 9 association studies between rs11066301 and risk of CAD. WTCCC (Wellcome Trust Case Control Consortium), GerMIFSI (German MI Family Study I), GerMIFSII (German MI Family Study II), MIGen (Myocardial Infarction Genetics Consortium), COROGENE (Corogene study), PennCATH (PennCATH), Medstar (MedSTAR), OHGS (Ottawa Heart Genomics Study).

**Figure 5:**
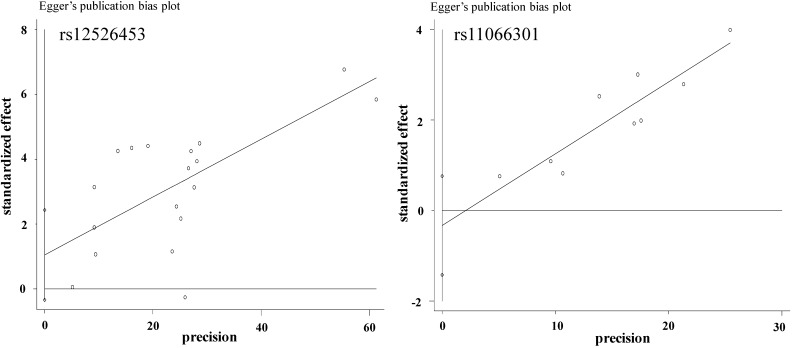
Egger’s publication bias plot for the relationship between the two SNPs and CAD in the meta-analyses.

Seven studies including 9 stages were included in the meta-analysis of *PTPN11* rs11066301, including 13,618 CAD cases and 13,479 controls from 2 ethnicities (Europeans and Asians). The pooled data showed a significant association between rs11066301 and CAD risk (OR = 1.15, 95% CI = 1.11–1.20, *p* < 0.0001, fixed-effects method, Figure 4). No heterogeneity was found in this meta-analysis (I^2^ = 0.0%, *p* = 0.985). No visual publication bias in the meta-analysis was detected by the funnel plot (Figure 5).

**Table 1:** Genotype and allele frequencies in SNPs in cases and controls.

SNP/Group	Genotype			χ^2^	P (df = 2)	Allele		χ^2^	P (df = 1)	HWE	OR (95% CI)
rs12526453	CC (%)	CG (%)	GG (%)			C (%)	G (%)				
case	594 (89.2)	72 (10.8)	0 (0.0)			1260 (94.6)	72 (5.4)			0.25	
control	375 (89.3)	45 (10.7)	0 (0.0)	0.00	1.00	795 (94.6)	45 (5.4)	0.00	1.00	0.62	1.01 (0.69–1.48)
rs11066301	AA (%)	AG (%)	GG (%)			A (%)	G (%)				
case	606 (91.0)	54 (8.1)	6 (0.9)			1266 (95.0)	66 (5.0)			0.003	
control	378 (90.0)	36 (8.6)	6 (1.4)	0.74	0.69	792 (94.3)	48 (5.7)	0.60	0.44	0.06	1.16 (0.79–1.71)
rs3828329	CC (%)	CT (%)	TT (%)			C (%)	T (%)				
case	360 (54.1)	270 (40.5)	36 (5.4)			990 (74.3)	342 (25.7)			0.13	
control	270 (64.3)	138 (32.9)	12 (2.8)	12.48	**0.002**	678 (80.7)	162 (19.3)	11.80	**0.0006**	0.34	1.45 (1.17–1.78)

**Table 2:** Comparison of the dominant model and recessive model between cases and controls.

SNP/Group	Dominant		χ^2^	P (df = 1)	OR (95% CI)	Recessive		χ^2^	P (df = 1)	OR (95% CI)
rs12526453	CC	CG + GG				CC + CG	GG			
case	594	72				666	0			
control	375	45	0.00	1.00	1.01 (0.69–1.48)	420	0	NA	NA	NA
rs11066301	AA	AG + GG				AA + AG	GG			
case	606	60				660	6			
control	378	42	0.30	0.58	0.89 (059–1.39)	414	6	NA	NA	0.63 (0.20–1.96)
rs3828329	CC	CT + TT				CC + CT	TT			
case	360	306				630	36			
control	270	150	11.07	0.0009	1.53 (1.19–1.97)	408	12	3.96	0.047	1.94 (1.00–3.78)

**Table 3:** Association of *ACP1* rs3828329 with CAD in different gender.

Gender	Group	Genotype(n)	Allele(n)	Additive		Dominant		Recessive	
		CC/CT/TT	C/T	P (df = 1)	OR (95% CI)	P (df = 1)	OR (95% CI)	P (df = 1)	OR (95% CI)
male	cases (*N* = 400)	242/172/20	656/212	0.109	1.25 (0.95–1.65)	0.120	1.29 (0.93–1.80)	0.337	1.53 (0.64–3.68)
	controls (*N* = 252)	142/80/7	364/94						
female	cases (*N* = 261)	118/98/16	334/130	0.001	1.80 (1.29–2.50)	0.0008	1.96 (1.32–2.92)	0.044	2.76 (1.00–7.66)
	controls (*N* = 177)	128/58/5	314/68						

**Table 4:** *Post hoc* analysis of *ACP1* rs3828329 with the risk of CAD in different age subgroups.

Gender	Age	Group	Genotype(n)	X^2^	P (df = 2)	Allele(n)	X^2^	P (df = 1)	OR (95% CI)
			CC/CT/TT			C/T			
All	<65	cases (*N* = 338)	177/148/13			502/174			
		controls (*N* = 247)	154/88/5	6.41	0.041	396/98	5.57	0.018	1.40 (1.06–1.85)
	≥65	cases (*N* = 328)	183/122/23			488/168			
		controls (*N* = 173)	116/50/7	6.34	0.042	282/64	6.44	0.011	1.52 (1.10–2.10)
male	<65	cases (*N* = 202)	104/89/9			297/107			
		controls (*N* = 136)	84/49/3	3.99	0.136	217/55	3.50	0.061	1.42 (0.98–2.05)
	≥65	cases (*N* = 232)	138/83/11			359/105			
		controls (*N* = 91)	58/31/2	1.30	0.522	147/35	0.89	0.345	1.23 (0.80–1.88)
female	<65	cases (*N* = 136)	73/59/4			205/67			
		controls (*N* = 111)	70/39/2	2.30	0.316	179/43	1.96	0.161	1.36 (0.88–2.10)
	≥65	cases (*N* = 96)	45/39/12			129/63			
		controls (*N* = 80)	58/19/3	12.58	0.002	135/25	10.56	0.001	2.27 (1.37–3.75)

### 2.3. Case-Control Study

A case-control cohort was recruited to investigate the relationships between three SNPs and CAD risk in Han Chinese. The distribution of genotypes and alleles were listed in Table 1. No departure from HWE was observed for all three SNPs in controls (*p* ≥ 0.05). Among the studied SNPs, only rs3828329 of the *ACP1* gene was significantly correlated with CAD risk (Table 1, genotype: χ^2^ = 12.48, df = 2, *p* = 0.002; allele: χ^2^ = 11.80, df = 1, *p* = 0.0006, OR = 1.45, 95% CI = 1.17–1.78). Moreover, rs3828329 showed a strong association with CAD risk under the dominant and recessive model (Table 2, dominant: OR = 1.53, 95% CI = 1.19–1.97, *p* = 0.0009; recessive: OR = 1.94, 95% CI = 1.00–3.78, *p* = 0.047). No significant differences between case and control groups were found for rs12526453 and rs11066301 (Table 1 and Table 2). Additionally, we performed a gender-stratified comparison between cases and controls with respect to three genetic models (including additive model, dominant model and recessive model). Significant associations were found in all three genetic models in the females (Additive model: *p* = 0.001, OR = 1.80, 95% CI = 1.29–2.50; dominant model: *p* = 0.03, OR = 1.69, 95% CI = 1.04–2.75; recessive model: *p* = 0.0008, OR = 1.96, 95% CI = 1.32–2.92, Table 3). A further subgroup analysis for age showed significant association of rs3828329 with CAD in persons younger than 65 years (Table 4, *p* = 0.018, OR = 1.40, 95% CI = 1.06–1.85) and over 65 years older (Table 4, *p* = 0.011, OR = 1.52, 95% CI = 1.10–2.10). A significant association was observed between rs3828329 and CAD risk in females older than 65 years (genotype: χ^2^ = 12.58, df = 2, *p* = 0.002; allele: χ^2^ = 10.56, df = 1, *p* = 0.001, OR = 2.27, 95% CI = 1.37–3.75).

## 3. Discussion

In this work, the significance of the polymorphisms of the three phosphatase association genes (*PHACTR1*, *ACP1* and *PTPN11*) in CAD was explored through meta-analysis and case-control study. Our results showed that rs12526453 of the *PHACTR11* gene and rs11066301 of *PTPN11* gene were associated with CAD risk in multiple populations. The rs3828329 of the *ACP1* gene is closely related to CAD risk in Han Chinese. In addition, through gender-stratified comparison, rs3828329 of the *ACP1* gene was associated with CAD risk in females. 

Age is a predictor of CAD [30]. The incidence of cardiovascular disease in older persons above 65 years is about 80% higher than that in the younger persons [31]. Persons aged 65 years or older constitute a growing proportion of the whole population and have higher cardiovascular morbidity and mortality. The optimal strategy to predict the risk of cardiac events in this group remains unknown [32]. Gender difference is often observed in the prevalence and clinical outcomes of human diseases [33], and there is a higher prevalence of cardiovascular disease in females [34]. Previous studies have revealed the different CAD risks in females and males in Han Chinese [35]. Zhang *et al.* [36] suggested that the *LPA* gene rs7767084-CC was a protective factor against CAD only in females. Peng *et al.* [37] revealed the significant association between the *KIF6* variant and CAD in women rather than in men. The *ACP1* genetic polymorphism is associated with a CAD risk in females with diabetes [38]. Our results suggested a significant association of *ACP1* rs3828329 with CAD in females aged 65 years and older. Specifically, rs3828329-T carriers had a 227% increased risk of CAD in the female subjects aged 65 and over. This can be partly explained by the particular genetic backgrounds and dietary habits of the Chinese. Our findings provide new clues to predict the risk of cardiac events in older female population. 

Phosphatase is implicated in many human diseases, such as cancer, diabetes and cardiovascular disease [39,40]. Recent study [41] revealed that serum alkaline phosphatase is an independent predictor of mortality, myocardial infarction, or stent thrombosis in CAD patients after percutaneous coronary intervention (PCI) with a drug-eluting stent (DES). In addition to the three genes studied in this work, there are other phosphatase related genes implicated in CAD, for instance, the polymorphisms of phosphatase and tensin homologue (*PTEN*) gene are significantly associated with atherosclerotic cerebral infarction (ACI) in the Chinese population [42]. Genetic polymorphisms of the protein tyrosine phosphatase non-receptor 22 (PTPN22) are reported to be involved in atherosclerosis and played a role in the immune response involved in the pathogenesis of CAD [43,44]. The polymorphism of protein tyrosine phosphatase 1B (PTP1B) is associated with a decreased risk of CAD in the Han Chinese population [45], and the phosphodiesterase-1 (PC-1) variant was reported to be associated with metabolic syndrome in patients with CAD [46].

Common variants of *ACP1* were shown to be associated with the risk of multiple diseases, such as favism [47], cancer [48], type 1 diabetes (T1D) [49] and cardiovascular events [38]. The *ACP1* polymorphism may play an important role in CAD through inducing enzymatic activity and affecting biochemical and functional properties of p53 [15]. SNP rs3828329 on 2p25.3 in the *ACP1* gene is has been shown to be significantly associated with fasting insulin and insulin sensitivity in type 2 diabetes in Mexican-Americans [50]. However, there is little evidence for the association between *ACP1* rs3828329 and CAD risk. In this work, our case-control study showed that rs3828329 was significantly associated with CAD in Han Chinese. A statistical calculation showed that the rs3828329 of the *ACP1* gene had 93.9% power to detect the relative risk at the nominal Type I error rate <0.05. The sample size may not be optimal, although it should be sufficient to describe a tendency that may guide clinical practice.

*PHACTR1* variants that influence the risk of myocardial infarction (MI) in genome-wide association studies (GWAS) were identified [51]. SNP rs12526453 on chromosome 6p24.1 located in *PHACTR1* showed directionally consistent associations with CAD risk in type 2 diabetes [52]. The other variant rs9349379 located in the intronic region of *PHACTR1* was shown to be the same background haplotype as rs12526453 (D’ = 0.98, r^2^ = 0.37) [10]. Both the two SNPs are strongly associated with coronary artery calcification (CAC) [53] and coronary artery stenosis [10]. Epidemiological studies demonstrated that rs12526453 is strongly connected with early-onset MI risk in both European and South Asian populations [54]. In the following Korean study, no significant association was found between rs12526453 and CAD risk [55]. Through the case-control study of Eastern Chinese, no convincing evidence was found for the relationship between rs12526453 and CAD risk. SNPs in the *PTPN11* locus are considered to be a cause of Noonan syndrome and LEOPARD syndrome [56,57]. Evidence has confirmed that *PTPN11* mutations are correlated with congenital heart defects in Noonan and LEOPARD syndromes [58]. The *PTPN11* polymorphism rs11066301 located on 12q24 was shown to be associated with platelet count in humans [59]. The *PTPN11* polymorphisms could influence serum lipid levels in a sex-specific pattern in Northeast Chinese [60]. In this work, we focused on the relation between rs11066301 and CAD in Eastern Chinese population. The association between rs11066301 and CAD risk was not found in this work. In the case-control study, rs11066301 and rs12526453 had less than 15% power to detect the relative risk. The negative results of the two SNPs may be due to lack of power, and it may also be attributed to the different genotype and allele frequencies of these SNPs in subjects with the particular genetic background and the Chinese lifestyle. The allele frequencies of rs11066301-G and rs12526453-G in HapMap data showed that there were huge ethnic differences between Asian (0.0% and 0.0%) and European (35.8% and 36.7%) populations. This is in agreement with our observation and indicates a very rare frequency in Han Chinese (rs11066301-G allele frequency = 5.7% and rs12526453-G allele frequency = 5.4%, respectively). However, discordant results were obtained for rs11066301 and rs12526453. This may be due to the lack of statistical power for some studies or genetic heterogeneity in the genes. Our meta-analysis of 118,256 individuals suggested that rs12526453 may predict a 14% increased risk of CAD in multiple populations (OR = 1.14, *p* < 0.0001). The rs11066301 showed a significant association with CAD risks in European and Asian populations through meta-analysis of 27,097 individuals. Strong heterogeneity in the meta-analysis for rs12526453 was mostly due to the different populations in the European studies and the Asian studies (Table 5).

There are several limitations of this work. Firstly, the sample size of the case-control study was moderate and might not be sufficient to perceive genes involved with moderate or minor effect. Secondly, only three SNPs were checked for the association with CAD. Other genetic polymorphisms in the selected genes might be involved as the real functional markers of CAD. Further large sample studies are necessary for verifying the association between other polymorphisms of phosphatase and CAD risk in various populations.

## 4. Experimental Section

### 4.1. Literature Review and Data Extraction

The meta-analyses were performed to examine the association of the three SNPs with CAD. We systematically searched in multiple literature databases including EMBASE, PubMed, Web of Science and China National Knowledge Infrastructure (CNKI), Wanfang Chinese Periodical Database. The search strategy was to use the following keywords in different combinations: “*PHACTR1*”, “*PTPN11*”, “*ACP1*”, “polymorphism”, “variation”, “rs11066301”, “rs12526453”, “rs3828329” paired with “coronary artery disease”, respectively. Full text articles were read to select the related information. References listed on the retrieved articles and previous meta-analyses on this topic were searched to appraise other studies of potential relevance. Meanwhile, the papers published in Chinese or English till 2013 were included. Studies were selected based on the following inclusion criteria: (1) the study must be case-control or a prospective design; (2) the study must evaluate the relationship between *ACP1* rs3828329, *PHACTR1* rs12526453 or *PTPN11* rs11066301and CAD; (3) the study contains complete data with genotype and allele frequencies or odds ratio (OR) with 95% confidence interval (95% CI); (4) the genotype distribution of controls is in Hardy-Weinberg equilibrium (HWE). Information was collected from each study, including first author, publication year, study design, total number of cases and controls, OR and 95% CI.

**Table 5:** Summary estimates of the OR of rs12526453 polymorphism in subgroup analyses.

Study Population	Studies, n	OR (95% CI)	Weight %	Z	P(z)	I^2^	*p*	τ^2^	Heterogeneity Statistic
European	7	1.14 (1.09–1.19)	45.75	5.60	<0.0001	67.1%	0.006	0.0023	18.24
North American	4	1.18 (1.06–1.31)	9.97	3.14	0.002	35.6%	0.199	0.0041	4.66
Asian	7	1.14 (1.08–1.21)	34.59	4.58	<0.0001	67.6%	0.005	0.0034	18.52
European and South Asian	1	1.13 (1.09–1.17)	9.69	6.76	<0.0001	NA	NA	0.00	0.00
Overall	19	1.14 (1.11–1.17)	100.00	9.37	<0.0001	57.7%	0.001	0.0016	42.53

### 4.2. Case-Control Study Sample Collection

A total of 1086 unrelated individual inpatients were recruited from LaiWu TaiShan Medical College Hospital in Laiwu city of Shandong province, China. Among them, 666 CAD patients were confirmed by the angiographic evidence that the stenosis was greater than 50% in at least the major coronary artery [61] or there was a history of prior angioplasty, or a history of coronary artery bypass surgery. The 420 controls were selected from a well-characterized random sample of the Ximen Community residents in Laiwu city. All individuals did not have any atherosclerotic vascular disease. All the subjects were unrelated Han Chinese and did not have any severe liver or kidney disease or congenital heart disease or cardiomyopathy. Patients were diagnosed by standardized coronary angiography according to the Seldinger’s method [62], and judged by at least two independent cardiologists. The study was approved by the Ethical Committee of TaiShan Medical College Hospital. Informed written consents were obtained from all subjects.

### 4.3. SNP Genotyping

Human genomic DNA was isolated from peripheral blood samples using a conventional phenol/chloroform method, and was quantified using the PicoGreen^®^ double strand (dsDNA) DNA Quantification Kit (Molecular Probes, Inc., Eugene, OR, USA). Amplification was performed on the ABI Geneamp^®^ PCR System 9700 Dual 384-Well Sample Block Module (Applied Biosystems, Foster City, CA, USA) for the Polymerase Chain Reaction (PCR). PCR conditions included an initial denaturation stage at 94 °C for 15 s, followed by 45 amplification cycles of 94 °C for 20 s, 56 °C for 30 s, and primer extension at 72 °C for 1 min, and then a final extension for 3 min at 72 °C. Primer extension for genotyping was performed on the SEQUENOM^®^ Mass-ARRAY iPLEX^®^ platform according to the manufacturer's instructions [63]. 

### 4.4. Statistical Analysis

The meta-analyses were performed by the RevMan software (version 5.1, Cochrane Collaboration, Oxford, UK) and the Stata software (version 11.0, Stata Corporation, College Station, TX, USA). Statistical heterogeneity between studies was estimated using the Q-test [64] and inconsistency index (I^2^ statistic) [64]. An I^2^ value >50% indicated a significant heterogeneity among the studies included in the meta-analysis [64]. Random-effects model based on the inverse-variance method was used for the studies with high heterogeneity, and for others, fixed-effects method was applied [65]. Publication bias was detected by the funnel plots and Egger regression test [66]. 

For the case-control study, HWE was analyzed using Arlequin program (version 3.5) [67]. Differences in the genotype and allele frequencies between two groups were determined by CLUMP22 software with 10,000 Monte Carlo simulations [67]. Power analysis was performed using the Power and Sample Size Calculation software (v3.0.43). A two-sided *p*-value 0.05 was considered as significant.

## 5. Conclusions

Our case-control study showed that the rs3828329 of the *ACP1* gene is strongly associated with the CAD risk in Han Chinese females aged 65 years and older. Meta-analyses supported that the rs12526453 of *PHACTR11* gene and the rs11066301 of *PTPN11* gene are associated with CAD risk in multiple populations.
